# Late initiation of antiretroviral therapy: inequalities by educational level despite universal access to care and treatment

**DOI:** 10.1186/s12889-021-10421-8

**Published:** 2021-02-19

**Authors:** Amanda Rodrigues, Claudio J. Struchiner, Lara E. Coelho, Valdilea G. Veloso, Beatriz Grinsztejn, Paula M. Luz

**Affiliations:** 1 Escola Nacional de Saúde Pública, Fundação Oswaldo Cruz, Rio de Janeiro, Brazil; 2 Escola de Matemática Aplicada, Fundação Getúlio Vargas, Praia de Botafogo, 190, Rio de Janeiro, Brazil; 3 Instituto Nacional de Infectologia Evandro Chagas, Fundação Oswaldo Cruz, Av. Brasil 4365, Manguinhos, Rio de Janeiro, 21040-900 Brazil

**Keywords:** HIV, ART or antiretroviral therapy, Late treatment initiation, Universal care, Brazil

## Abstract

**Background:**

Late antiretroviral treatment initiation for HIV disease worsens health outcomes and contributes to ongoing transmission. We investigated whether socioeconomic inequalities exist in access to treatment in a setting with universal access to care and treatment.

**Methods:**

This study investigated the association of educational level, used as a proxy for socioeconomic status, with late treatment initiation and treatment initiation with advanced disease. Study participants included adults (≥25 years) who started treatment from 2005 to 2018 at Instituto Nacional de Infectologia Evandro Chagas of Fundação Oswaldo Cruz (INI/FIOCRUZ), Rio de Janeiro, Brazil. Educational level was categorized following UNESCO’s International Standard Classification of Education: incomplete basic education, basic education, secondary level, and tertiary level. We defined late treatment initiation as those initiating treatment with a CD4 < 350 cells/mL or an AIDS-defining event, and treatment initiation with advanced disease as those initiating treatment with a CD4 < 200 cells/mL or an AIDS-defining event. A directed acyclic graph (DAG) was constructed to represent the theoretical-operational model and to understand the involvement of covariates. Logistic regression models were used to estimate the adjusted odds ratios (aOR) and 95% confidence intervals (95%CI). Multiple imputation using a chained equations approach was used to treat missing values and non-linear terms for continuous variables were tested.

**Results:**

In total, 3226 individuals composed the study population: 876 (27.4%) had incomplete basic education, 540 (16.9%) basic, 1251 (39.2%) secondary level, and 525 (16.4%) tertiary level. Late treatment initiation was observed for 2076 (64.4%) while treatment initiation with advanced disease was observed for 1423 (44.1%). Compared to tertiary level of education, incomplete basic, basic and secondary level increased the odds of late treatment initiation by 89% (aOR:1.89 95%CI:1.47–2.43), 61% (aOR:1.61 95%CI:1.23–2.10), and 35% (aOR:1.35 95%CI:1.09–1.67). Likewise, the odds of treatment initiation with advanced disease was 2.5-fold (aOR:2.53 95%CI:1.97–3.26), 2-fold (aOR:2.07 95%CI:1.59–2.71), 1.5-fold (aOR:1.51 95%CI:1.21–1.88) higher for those with incomplete basic, basic and secondary level education compared to tertiary level.

**Conclusion:**

Despite universal access to HIV care and antiretroviral treatment, late treatment initiation and social inequalities persist. Lower educational level significantly increased the odds of both outcomes, reinforcing the existence of barriers to “universal” antiretroviral treatment.

## Background

A consensus definition of ‘late presentation’ was reached in October 2009 by the European study group for the purpose of identifying untreated HIV-infected individuals at an advanced stage of the disease [1]. Two definitions were put forth: late presenters as those “presenting for care with a CD4<350 cells/mL or with an AIDS-defining event, regardless of the CD4 cell count” and presentation with advanced HIV disease as those “presenting for care with a CD4<200 cells/mL or with an AIDS-defining event, regardless of the CD4 cell count”. In a large study from the Collaboration of Observational HIV Epidemiological Research in Europe (COHERE) including more than 84,000 individuals living with HIV from 35 European countries and following the consensus definition mentioned above, late presentation decreased over time in both Central and Northern Europe, especially among men who have sex with men (MSM). However, it increased for male intravenous drug users (IDUs) and female heterosexuals from Southern Europe and IDUs from Eastern Europe. Late presentation was associated with an increased rate of AIDS/deaths, particularly in the first year after HIV diagnosis, with significant variation across Europe [2].

In settings where antiretroviral therapy (ART) is available free-of-charge such as in Brazil, one may extend these definitions from presentation to care to treatment initiation. In Brazil, ART (initial as well as salvage regimens) has been provided free-of-charge since 1996, when provision was instituted by law, along with virologic and immunologic monitoring, and genotype testing upon virologic failure [3]. Brazilian treatment guidelines, which have been regularly released with updated treatment recommendations, increased the CD4 cell count threshold for treatment initiation, which was 200 cells/mL since 2001, to 350 cells/mL in 2004, and to 500 cells/mL in 2008. In 2013, guidelines were changed to recommend treatment to all individuals living with HIV irrespective of CD4 cell count [3]. In this scenario, the monitoring of how late individuals initiate treatment speaks to the success of HIV care in Brazil and other settings with universal provision of ART. Prior studies from Brazil suggest, again, analogously to results regarding late presentation to care conducted elsewhere, that late treatment initiation is pervasive [4–7].

Late presentation to HIV care coupled with late treatment initiation in the course of HIV disease progression worsens the health outcomes for individuals and can compromise the fight against the HIV/AIDS epidemic. For people living with HIV, late treatment initiation is associated with clinical progression, increased AIDS and non-AIDS related morbidity and mortality, higher propensity for treatment related adverse events and lower chances of achieving viral suppression [8–14]. At the population level, late treatment initiation implies potential onward viral transmission from unsuppressed HIV viral load among those living with HIV [15]. In 2013, The World Health Organization (WHO)‘s guidelines introduced the concept of HIV elimination through the “Test and Treat” strategy, that fosters prompt initiation of ART to all people living with HIV, irrespective CD4 cell count [16]. The randomized controlled trial HPTN052 demonstrated that among couples where one partner is HIV-infected and the other HIV-uninfected, early use of ART by the HIV-infected partner decreased HIV transmission by 96% compared with delayed treatment initiation [15]. The evidence for the effectiveness of Treatment as prevention (TasP) was also shown by the PARTNER study, with zero transmissions between same-sex mixed status couples when the HIV-infected partner attained viral suppression [17]. Accordingly, increasing access and utilization of HIV prevention, care and treatment services at the onset of the infection, can reduce viral load in the community and improve clinical prognosis of people living with HIV.

Despite the existence of a universal health system, equitable socio-organizational and geographical accessibility are not guaranteed, and population groups with low socioeconomic status face different barriers to access care and are often associated with worse health outcomes [18]. We investigated the association between late treatment initiation and low socioeconomic status using educational level as a proxy for socioeconomic position. A previous study conducted in European countries showed that low education levels were strongly associated with delayed HIV diagnosis and delayed ART initiation, highlighting the existence of socioeconomic inequalities in health in nine HIV cohorts from six countries: Austria, France, Greece, Italy, Spain and Switzerland [19]. Our working hypothesis was that lower educational level would be linked to higher risk of late treatment initiation and treatment initiation with advanced disease. Understanding the role of socioeconomic status and its effect in health outcomes may help design and implement public actions aimed at remedying social disadvantages [20].

## Methods

### Study population

**Fig. 1:**
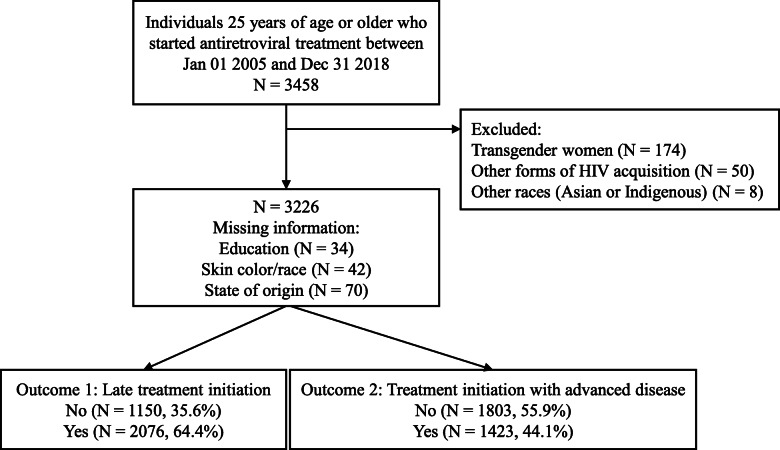
Flowchart of the study population

Recognized as a national reference center in clinical research and provision of primary, specialty and tertiary health services, Instituto Nacional de Infectologia Evandro Chagas of Fundação Oswaldo Cruz (INI/FIOCRUZ) has provided care to people living with HIV since 1986. INI has also sustained a longitudinal observational clinical database on individuals receiving HIV care that is updated regularly by trained staff and has been used in several studies [21–23]. For the present study, the study population included adults living with HIV who started treatment between Jan 1, 2005 and Dez 31, 2018. The initial year was determined based on Brazilian treatment guidelines which in late 2004 altered the recommended CD4 cell count threshold to 350 cells/L. As educational level was our primary exposure of interest, only adults aged 25 years or older were included to allow for completion of college education. For the present analysis, transgender women participants had to be excluded given that, through both the respondent driven sampling study [24] and the *Transcendendo* cohort [25], they were actively recruited for HIV testing and care at our institution, thus modifying the means of achieving our health care facility and, therefore the probability of late treatment initiation (Fig. 1). Additionally, due to low numbers, we excluded participants that had acquired HIV through mother-to-child transmission (*n* = 3), accidental exposure (*n* = 7), injection drug use (*n* = 21), and blood transfusion (*n* = 19); we also excluded participants of Asian or Indigenous origin (*n* = 8).

### Exposure

For the primary exposure of interest, educational level, we followed the UNESCO / International Standard Classification of Education (ISCED) and grouped educational level according to the following strata: (1) incomplete basic education comprising illiteracy and incomplete early childhood education, (2) basic education from sixth to ninth grade, (3) secondary level education that includes individuals who have completed high school and (4) tertiary level education comprising those with college or higher academic degree [19].

### Outcomes

For our outcomes of interest, we followed definitions for who should initiate antiretroviral treatment as suggested in the 2004 and 2008 Brazilian guidelines [3]. We defined late treatment initiation as those initiating treatment with a CD4 < 350 cells/mL or with an AIDS-defining event, regardless of the CD4 cell count, and treatment initiation with advanced disease as those initiating treatment with a CD4 < 200 cells/mL or with an AIDS-defining event, regardless of the CD4 cell count. CD4 cell counts performed within 6 months prior to up to 1 month after ART start date were considered, electing for the analyses the CD4 cell count that was closest to the ART start date. For participants that did not have a CD4 cell count within the stipulated range (*N* = 438), presence/absence of an AIDS-defining event, defined as the presence of an AIDS-defining event within 6 months prior to 6 months after ART initiation, was solely used to determine the presence of the outcomes of interest.

### Covariates

**Fig. 2:**
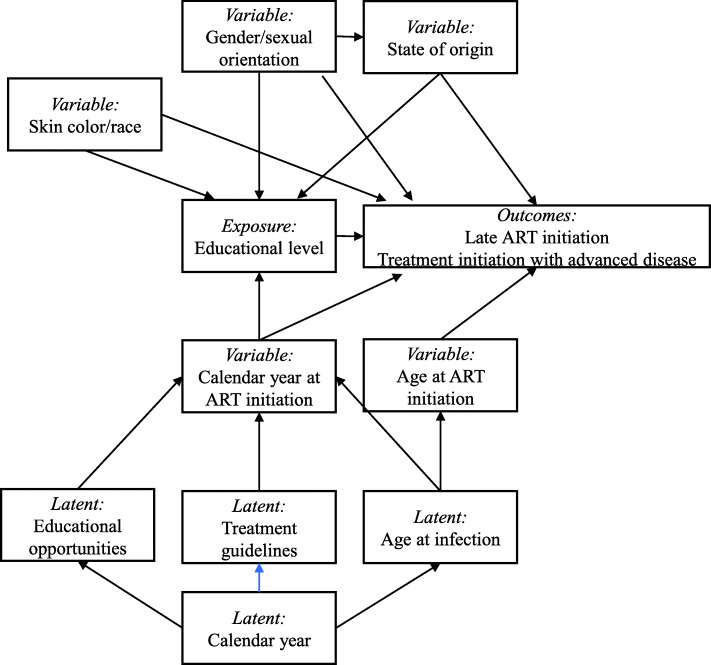
The proposed operational model depicting the relationship between exposure, outcome, and measured and latent variables

A directed acyclic graph (DAG) was constructed to represent the proposed operational model and to understand the involvement of covariates in the relationship between educational level and late treatment initiation/treatment initiation with advanced disease [26, 27]. In the construction of the DAG, based on authors’ conceptualization of the problem and published literature [4, 11, 19, 28, 29], we represented both objectively measured variables as well as latent variables as needed to adequately represent the relationship between the covariates. The DAG was constructed using the R package ‘dagitty’ [30] and was used to identify the minimum set of potential confounding variables. The objectively measured variables used in the DAG were gender/sexual orientation, skin color/race, state of origin (if from the State of Rio de Janeiro or not), calendar year at treatment initiation and age at treatment initiation. The latent factors that were presumed to influence year of treatment initiation which have themselves changed over the years were defined as educational opportunities, treatment guidelines and age at HIV infection. The gender/sexual orientation variable combines both the information of a person’s gender identity and sexual orientation as it pertains to the probable mode of HIV acquisition. In as much, the categories included cis-women, cis-men who have sex with men, heterosexual cis-men and other cis-men for whom their mode of HIV acquisition is unknown. The minimum set of potential confounding variables that needed to be controlled for to correctly measure the relationship between educational level and late treatment initiation/treatment initiation with advanced disease were gender/sexual orientation, skin color/race, state of origin and calendar year at treatment initiation (Fig. 2).

### Statistical analysis

Means and standard deviations, and absolute number and proportions were used to describe the characteristics of the study population according to the two outcomes of interest. Logistic regression models were used to quantify the association of educational level with the two outcomes using the set of adjustment variables as described above. We provide only the adjusted odds ratios (OR) and the respective 95% confidence intervals. Multiple imputation using a chained equations approach was used to impute the missing values in education (*n* = 34), skin color/race (*n* = 42), state of origin (*n* = 70). Also, we tested non-linear terms for the continuous variable “year of treatment initiation” using fractional polynomials but rejected the hypothesis that a non-linear association was present. The analyses were performed using R software, version 3.6.2, using the library “rms”, “Hmisc”, “epiDisplay” and “mfp”.

## Results

**Fig. 3:**
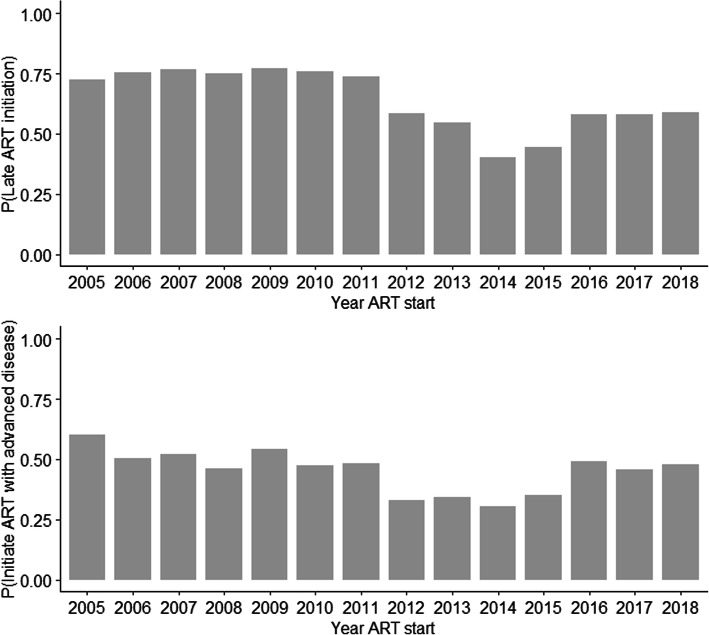
Percentage of individuals with late treatment initiation and treatment initiation with advanced disease by year, from 2005 to 2018

From January 01, 2005 to December 31, 2018, 3458 individuals 25 years of age or older initiated treatment at INI/FIOCRUZ. From these, 232 were excluded (details in the Methods, Fig. 1), resulting in a final study population of 3226 individuals. The educational level of these participants was: 876 (27.4%) had uncompleted basic education, 540 (16.9%) had basic education, 1251 (39.2%) had secondary level, and 525 (16.4%) tertiary level education (Table 1). Overall, mean age at ART initiation was 38.4 years (median 36.6, interquartile range 30.8–44.5), 29.1% were cis-women and 37.4% were cis-men who have sex with men. Most participants were non-white (21.9% black and 34.8% mixed [pardo]) and most were form the State of Rio de Janeiro (79.6%). Overall, late treatment initiation was observed for 2076 individuals (64.4%) while treatment initiation with advanced disease was observed for 1423 (44.1%). Both outcomes fluctuated over the years with the years 2012 to 2015 showing the lowest observed percentages (Fig. 3).

**Table 1:** Sociodemographic characteristics and adjusted odds ratios (95% confidence intervals) for the two outcomes: late treatment initiation and treatment initiation with advanced disease

		Late treatment initiation			Treatment initiation with advanced disease		
	Total	No	Yes	Odds Ratio (95%CI)	No	Yes	Odds Ratio (95%CI)
Total	3226	1150 (35.6)	2076 (64.4)		1803 (55.9)	1423 (44.1)	
Exposure of interest							
Educational level							
Tertiary	525 (16.4)	242 (46.1)	283 (53.9)	Ref.	366 (69.7)	159 (30.3)	Ref.
Secondary	1251 (39.2)	480 (38.4)	771 (61.6)	1.35 (1.09–1.67)	751 (60)	500 (40)	1.51 (1.21–1.88)
Basic	540 (16.9)	163 (30.2)	377 (69.8)	1.61 (1.23–2.1)	268 (49.6)	272 (50.4)	2.07 (1.59–2.71)
Incomplete basic	876 (27.4)	249 (28.4)	627 (71.6)	1.89 (1.47–2.43)	399 (45.5)	477 (54.5)	2.53 (1.97–3.26)
Covariates							
Age at treatment initiation							
Median (IQR)	36.6 (30.8,44.5)	35.5 (29.9,44)	37.2 (31.3,44.7)	–	35.5 (30.1,43.5)	38 (31.7,45.2)	–
Gender/Sexual orientation^a^							
Cis women	938 (29.1)	349 (37.2)	589 (62.8)	Ref.	559 (59.6)	379 (40.4)	Ref.
Cis MSM	1205 (37.4)	479 (39.8)	726 (60.2)	1.23 (1.01–1.49)	740 (61.4)	465 (38.6)	1.28 (1.06–1.55)
Cis heterosexual men	772 (23.9)	196 (25.4)	576 (74.6)	1.76 (1.42–2.18)	35 (43.4)	437 (56.6)	1.96 (1.61–2.39)
Cis men (unknown exposure)	311 (9.6)	126 (40.5)	185 (59.5)	1.18 (0.9–1.56)	169 (54.3)	142 (45.7)	1.62 (1.24–2.12)
Skin color/race							
White	1378 (43.3)	517 (37.5)	861 (62.5)	Ref.	816 (59.2)	562 (40.8)	Ref.
Black	697 (21.9)	217 (31.1)	480 (68.9)	1.34 (1.09–1.65)	347 (49.8)	350 (50.2)	1.34 (1.1–1.63)
Mixed (pardo)	1109 (34.8)	404 (36.4)	(63.6)	1.08 (0.91–1.28)	622 (56.1)	487 (43.9)	1.06 (0.9–1.26)
State of origin							
Rio de Janeiro	2511 (79.6)	914 (36.4)	1597 (63.6)	Ref.	1418 (56.5)	1093 (43.5)	Ref.
Other	645 (20.4)	212 (32.9)	433 (67.1)	1.07 (0.88–1.3)	347 (53.8)	298 (46.2)	1.02 (0.85–1.22)
Year of treatment initiation							
Median (IQR)	2011 (2008,2014)	2013 (2010,2015)	2011 (2008,2013)	0.86 (0.83–0.88)	2012 (2009,2014)	2011 (2008,2014)	0.94 (0.91–0.97)

*MSM* men who have sex with men, *RJ* Rio de Janeiro State, *ART* antiretroviral treatment, *CI* confidence interval

^a^ The gender/sexual orientation variable combines information of a person’s gender identity and sexual orientation as it pertains to the probable mode of HIV acquisition thus categorizing the study population into cis-women, cis-men who have sex with men, heterosexual cis-men and other cis-men for whom their mode of HIV acquisition is unknown

Educational level was significantly associated with late treatment initiation and with treatment initiation with advanced disease in a dose-response manner such that the lower the educational level, higher the odds of either outcome (Table 1). Having secondary level education compared to tertiary level increase the odds of late treatment initiation by 35%, while having basic education or incomplete basic education increase the odds of the outcome by 61 and 89%, respectively. Except for state of origin, the other covariates were also significantly associated with late treatment initiation with both cis MSM and heterosexual cis-men, compared to cis-women, having increased odds of late treatment initiation. Blacks were also at increase odds of late treatment initiation compared to whites.

When considering the second outcome of interest, treatment initiation with advanced disease, having secondary level education compared to tertiary level increased the odds of the outcome by 51%. The odds of treatment initiation with advanced disease was 2-fold and 2.5-fold higher among those with basic and incomplete basic education compared to those with tertiary education. Compared to women, men were significantly more likely to initiate treatment with advanced disease while blacks were also more likely to present with worse outcomes compared to whites. Finally, there was a significant inverse relationship with the covariate year of treatment initiation showing a decreasing trend in the prevalence of both outcomes over time.

## Discussion

Socioeconomic status is a multidimensional concept that encompasses educational level, occupation and income, in addition to subjective perceptions of social status [31]. In the present, we used educational level as a proxy for socioeconomic status to evaluate if the outcomes of interest occurred at different frequencies in population groups that differed in terms of educational level. Socio-economic status has been shown to play an important role in access to health services with previous studies reporting a positive association between higher educational level and early HIV diagnosis and ART initiation [19, 32, 33]. Our results show how low educational level is significantly associated with a higher odds of late treatment initiation and of treatment initiation with advanced disease in a dose-response manner, that is, the lower the educational level the higher the odds of either outcome. Having incomplete basic education increased the odds of late treatment initiation by 89% and increased the odds of treatment initiation with advanced disease by 2.5-fold. These findings highlight how, despite availability of free-of-charge ART, the HIV epidemic remains entrenched among the socially vulnerable who do not equally benefit from timely ART initiation. Furthermore, our findings show that socioeconomic inequalities are particularly visible when considering the most severe outcome, treatment initiation with advanced disease.

WHO’s “Test and Treat” strategy has not only advocated for the prompt initiation of ART after diagnosis of HIV-infection, but also proposed to optimize the quality of services along the continuum of care. Previous studies emphasize that the success of the strategy to increase ART coverage depends on the expansion of HIV screening programs to detect HIV infections already in course among those unaware of their infection [34]. Despite having a universal health system that offers ART to people living with HIV since 1996 as well as voluntary counselling and testing services across the country, ours and previous studies show that late treatment initiation is pervasive in Brazil. In a study that used Brazilian HIV surveillance data for the period 2003 to 2006, the prevalence of late entry, defined by the presence of an AIDS defining illness or a CD4 cell count ≤200 cells/mL or an HIV diagnosis at death, was reported at 44% [4]. In a more recent analysis from Goiania, a city in the central-west part of Brazil, that used data from 2009 to 2012, found that the prevalence of late initiation of ART, defined as CD4 cell count < 200 cells/mL or presence of AIDS-defining illness at treatment initiation or up to 30 days after initiation, was of 56% [7]. Globally, the situation is similar. In an analysis of data from 2002 to 2009, the immune status of participants initiating treatment in a clinic participating in a multicohort collaboration spanning 6 continents (International epidemiological Databases to Evaluate AIDS and ART Cohort Collaboration) remained low: median CD4 cell counts was ≤200 cells/mL in low-income countries and ≤ 300 cells/mL in high-income countries [35]. Similarly, in another analysis of over 84,000 individuals from 23 countries in Europe presenting for HIV care between 2000 and 2011, late presentation, defined as HIV diagnosis with a CD4 count < 350/mm3 or an AIDS diagnosis within 6 months of HIV diagnosis, was observed for 54% [2]. Our results, which rely on data from 2005 to 2018, still finds that late treatment initiation is highly prevalent, occurring in 64% of participants, while 44% initiate treatment with advanced disease, and thus reaffirms the continued need to identify individuals for timely treatment initiation.

Notably, as mentioned in the Background, there is overwhelming evidence that socioeconomic status significantly influences development and prevalence of health conditions. In our study, the gradient of the association between educational level and poor outcomes is evident. Late treatment initiation was observed among 71, 69, 61 and 53% of participants with uncompleted basic, basic, secondary, and tertiary education, respectively. When considering the most extreme outcome of treatment initiation with advanced disease, 55, 50, 40, and 30% of participants with uncompleted basic, basic, secondary, and tertiary education, respectively, presented with the outcome. These results translate into a two-fold increased odds of treatment initiation with advanced disease among those with basic education compared to tertiary level. Similar findings have been reported in Spain, where 56% of over 1000 individuals initiated treatment late and socioeconomic status was found associated with the outcome [36]. A study of nine European countries with universal healthcare systems also showed that those with lower educational level did not equally benefit from timely antiretroviral treatment initiation [19]. Moreover, in a study of individuals diagnosed with acute HIV-infection at a Montreal primary clinic (The Montreal Primary HIV Infection Study), having a paid employment, another proxy for socioeconomic status, was also found to influence the timely initiation of treatment (defined as treatment initiated within 180 days of the HIV diagnosis) in this high-income country with universal access to care [37]. Taken together, these results highlight the need to promote early treatment initiation for all. Moreover, the observed inequitable provision of treatment requires addressing structural and societal factors that are the fundamental causes of health inequality, from poverty, to racism, stigma towards gender and sexual minorities as well as stigma towards people living with HIV [38–40]. Finally, studies from Brazil have shown that willingness to use pre-exposure prophylaxis was associated with proxies of socioeconomic status [41], suggesting that the inequitable provision of health services extends to prevention efforts.

Our results also show that blacks when compared to whites and men when compared to women had increased odds of late treatment initiation and treatment initiation with advanced disease. In particular, heterosexual cis-men had an almost two-fold increase in the odds of treatment initiation with advanced disease. Stigma and discrimination surrounding race, gender identity and sexual orientation can directly impact the fight against the HIV epidemic [42, 43]. Accordingly, we interpret our findings as resulting from the persistent effects of stigma and discrimination, pervasive in Brazilian society [44]. In a study conducted in a major city in the Southeast of Brazil, blacks had 50% higher odds of experiencing discrimination than whites, even after controlling for income, education, social status, and health problems [45]. Moreover, HIV-related stigma has been found to represent a barrier to HIV testing among MSMs in a metropolitan area in Brazil [46]. Importantly, prior studies from our cohort show that both MSM and heterosexual men have twice the risk of AIDS-related mortality compared to women [29]; a finding that likely is interconnected with the these presented in this analysis. When comparing the two outcomes studied here, outcomes were worse among heterosexual men than MSM, which is in accordance to a Swiss study [47] and may be due to MSM being more aware of HIV infection and consequently more attuned to testing, prevention and treatment services.

Our study has limitations that should be acknowledged. Though socioeconomic status is a multidimensional concept that extends beyond educational level, the use of educational level as an indicator of socioeconomic status in adult populations has been shown to be one of the indicators most capable of capturing aspects related to an individual’s lifestyle as health behaviors and less likely to be affected by diseases in adulthood when compared to income and occupation [48]. The differential access of transgender women to our health care center through recent studies such as *Transcender* precluded us to include this population and as such limit the generalizability of the findings. Similarly, individuals who acquired HIV from injection drug use, transfusion, and other routes, in addition to those of Asian (*N* = 5) and indigenous (*N* = 3) race/skin color were excluded due to low number with the caveat that results cannot be extrapolated to these populations.

## Conclusion

Our results show that despite universal access to HIV care and treatment, late treatment initiation and social inequalities persist. Lower educational level significantly increased the odds of both outcomes, reinforcing the existence of barriers to “universal” antiretroviral treatment. Individuals with low educational level were more likely to initiate treatment late or with advanced disease, and the strength of the association was even greater for the latter, more extreme outcome. Furthermore, the increased odds of both outcomes among blacks and both cisgender MSM and heterosexual men draws attention to the need of programs and policies that reach different races and genders, in attempt to overcome social barriers.

## Data Availability

The datasets during and/or analyzed during the current study available from the corresponding author on reasonable request.
