# Correction to: Late initiation of antiretroviral therapy: inequalities by educational level despite universal access to care and treatment

**DOI:** 10.1186/s12889-021-10683-2

**Published:** 2021-04-01

**Authors:** Amanda Rodrigues, Claudio J. Struchiner, Lara E. Coelho, Valdilea G. Veloso, Beatriz Grinsztejn, Paula M. Luz

**Affiliations:** 1 Escola Nacional de Saúde Pública, Fundação Oswaldo Cruz, Rio de Janeiro, Brazil; 2 Escola de Matemática Aplicada, Fundação Getúlio Vargas, Praia de Botafogo, 190, Rio de Janeiro, Brazil; 3 Instituto Nacional de Infectologia Evandro Chagas, Fundação Oswaldo Cruz, Av. Brasil 4365, Manguinhos, Rio de Janeiro, 21040-900 Brazil

**Correction to: BMC Public Health 21, 389 (2021)**

**https://doi.org/10.1186/s12889-021-10421-8**

It was highlighted that in the original article [1] the Acknowledgments section was incorrect. This Correction article shows the incorrect and correct Acknowledgments section. The original article has been updated.

**Incorrect**

Acknowledgements

Not applicable.

**Correct**

Acknowledgements

This study was financed in part by the Coordination for the Improvement of Higher Education Personnel (Coordenação de Aperfeiçoamento de Pessoal de Nível Superior [CAPES]), finance Code 001.
